# Lipid–peptide bioconjugation through pyridyl disulfide reaction chemistry and its application in cell targeting and drug delivery

**DOI:** 10.1186/s12951-019-0509-8

**Published:** 2019-06-21

**Authors:** Diego de la Fuente-Herreruela, Ajay K. Monnappa, Mónica Muñoz-Úbeda, Aarón Morallón-Piña, Eduardo Enciso, Luis Sánchez, Fabrice Giusti, Paolo Natale, Iván López-Montero

**Affiliations:** 1 Dto. Química Física, Universidad Complutense de Madrid, Avenida Complutense s/n, 28040 Madrid, Spain; 2 Instituto de Investigación Hospital Doce de Octubre (i+12), Avenida de Córdoba s/n, 28041 Madrid, Spain; 3 Department of Biological Sciences, School of Life Sciences, Ulsan National Institute of Science and Technology (UNIST), Ulsan, 689-798 Republic of Korea; 4 Dto. Química Orgánica, Universidad Complutense de Madrid, Avenida Complutense s/n, 28040 Madrid, Spain; 5 Institut de Chimie Séparative de Marcoule, ICSM, UMR 5257, Site de Marcoule-Bât, 426 BP 17 171, 30207 Bagnols sur Ceze, France

**Keywords:** Smart liposomes, Disulfide bonds, Targeting peptide, GALA, Endosomal escape

## Abstract

**Background:**

The design of efficient drug delivery vectors requires versatile formulations able to simultaneously direct a multitude of molecular targets and to bypass the endosomal recycling pathway of cells. Liposomal-based vectors need the decoration of the lipid surface with specific peptides to fulfill the functional requirements. The unspecific binding of peptides to the lipid surface is often accompanied with uncontrolled formulations and thus preventing the molecular mechanisms of a successful therapy.

**Results:**

We present a simple synthesis pathway to anchor cysteine-terminal peptides to thiol-reactive lipids for adequate and quantitative liposomal formulations. As a proof of concept, we have synthesized two different lipopeptides based on (a) the truncated Fibroblast Growth Factor (tbFGF) for cell targeting and (b) the pH sensitive and fusogenic GALA peptide for endosomal scape.

**Conclusions:**

The incorporation of these two lipopeptides in the liposomal formulation improves the fibroblast cell targeting and promotes the direct delivery of cargo molecules to the cytoplasm of the cell.

**Electronic supplementary material:**

The online version of this article (10.1186/s12951-019-0509-8) contains supplementary material, which is available to authorized users.

## Background

Liposomes have been extensively used as delivery vectors for pharmaceuticals as they have a series of advantages over other molecular release systems [1]. Liposomes are non-toxic, and completely biodegradable and do not exhibit immunogenicity [2]. Their phospholipid bilayer envelope provides both hydrophobic and hydrophilic moieties for a diverse kind of active cargo molecules, increasing their stability and reducing their toxicity. In addition, liposomes are versatile scaffolds with tunable physicochemical properties. The thousands of lipid species [3] can be used to tailor the specific requirements for improved delivery. Spontaneous curvature [4], bending rigidity [5], dilational elasticity [6], membrane fluidity [7] or surface charge [1] are easily controlled through the lipid composition.

The controlled and selective delivery of compounds into cells is a key element of targeted drug delivery therapies. Major innovations in liposome technology were achieved by triggered-release strategies using activating sources such as pH, ultrasound, heat or light [8, 9]. The drug release can be time regulated and locally restricted to specific sites with suitable formulations including externally switchable molecules. In practice, they are, however, been difficult to engineer. The new generation of smart liposomes takes advantage instead of well-controlled biochemical switches already provided by the targeted cells. This implies the surface modification of liposomes with passive or active targeting approaches and improved intracellular delivering systems [1].

A paradigmatic strategy of passive targeting consists on the incorporation of PEGylated lipids in the liposomal composition to avoid the detection by the host immune system [10]. PEG-grafted liposomes improve the residence time in the blood circulation, as compared to conventional liposomes [11]. Active targeting is based on cellular receptors that are found on surface of the target cell and demands the modification of the liposomal surface with specific molecules able to recognize or bind the present surface receptors. After cellular uptake, liposomes are usually trapped in endosomes [12, 13] and not able to release their therapeutic cargos within the cell. Numerous formulations take advantage of the acidic medium of the endosomes and include specialized pH-dependent fusion peptides to promote endosomal escape [14–16]. Combined strategies improve simultaneously the ability of liposomes to accumulate on the target cell and the uptake of the active drug in the lumen of the cell [17].

The surface functionalization of liposomes is an important step to improve their delivery efficiency. The very reactive sulfhydryl group of cysteine amino acid residues allows the conjugation of cysteine containing-peptides through classical sulfhydryl-reactive crosslinkers or thiol reagents. The thiol is susceptible to oxidation promoting the formation of a thioether or disulfide bonds. The first reaction can be achieved with high concentration of maleimides following a Michael addition reaction to form succinimide thioethers derivatives or in the presence of haloacetyls (iodoacetimide) reagents following nucleophilic substitution [18]. However, the succinimide bond can be hydrolyzed spontaneously thus losing the binding between the peptide and the ligand [19]. Peptide bioconjugation based on the formation of disulfide bonds, where the cysteine groups react with pyridyl disulfides reagents, produces a labile disulfide bond in both redox and hydrolytic conditions but only potentially cleaved due to action of thioreductases enzymes in biological contexts [20].

Here we present a straightforward synthesis pathway to anchor peptides with terminal cysteine residues to sulfydryl-reactive lipids for adequate and quantitative liposomal formulations. Based in the pyridyl disulfide reaction chemistry [21], we have functionalized lipids with (a) a truncated Fribloblast Growth Factor (FGF) for cell targeting and (b) a pH sensitive fusogenic peptide (GALA) for endosomal escape. The basic fibroblast growth factor (bFGF) is one of the 23 multifunctional proteins belonging to the family of fibroblast growth factors that binds to the FGF surface membrane receptors (FGFRs) [22] and widely used as a targeting molecule due to its mitogenic, chemotactic and angiogenic activities promoting the rapid proliferation of cells. The truncated bFGF (tbFGF) is a 9-amino acid peptide that includes a cysteine in the carboxy terminal end (NH2-KRTGQYKLC-COOH) [23] and although it is able to bind to FGFRs it is not able to induce cell proliferation [24–26].

The GALA peptide (NH2-WEAA-LAEA-LAEA-LAE-H-LAEA-LAEA-LEALAA-COOH) is a member of the pH-sensitive peptide family [27, 28] and originates from the amino terminal segment of the H2A subunit of hemagglutinin from the influenza virus [25, 29, 30]. At low pH (i.e. pH = 5) the GALA peptide organizes into an amphipathic alpha helix partitioning the amino acid side chains into a hydrophilic or a hydrophobic surface. This reorganization promotes the self-oligomerization of 10 peptides [31] that is able to penetrate into the hydrophobic core of lipid membranes forming pores with a diameter of 5–10 Å [17, 25]. At physiological and basic pH (pH > 7) the GALA peptide presents a random coil configuration and exhibits no membrane activity [28]. Triggered by the acidic medium within the lumen of the endosomes, it has been shown that GALA effectively penetrates and permeates cell lipid bilayers and allows the endosomal escape during the internalization of drugs into the cytosol via endocytosis [28, 31].

As a proof of concept, our results show that the incorporation of both synthesized bioconjugated lipopeptides in liposomal formulations improved the cell targeting and promoted the direct delivery of cargo molecules in the cytosolic moiety of cultured mouse embryonic fibroblasts (MEFs).

## Results

### Lipid–peptide conjugation through pyridyl disulfide reaction chemistry

**Fig. 1:**
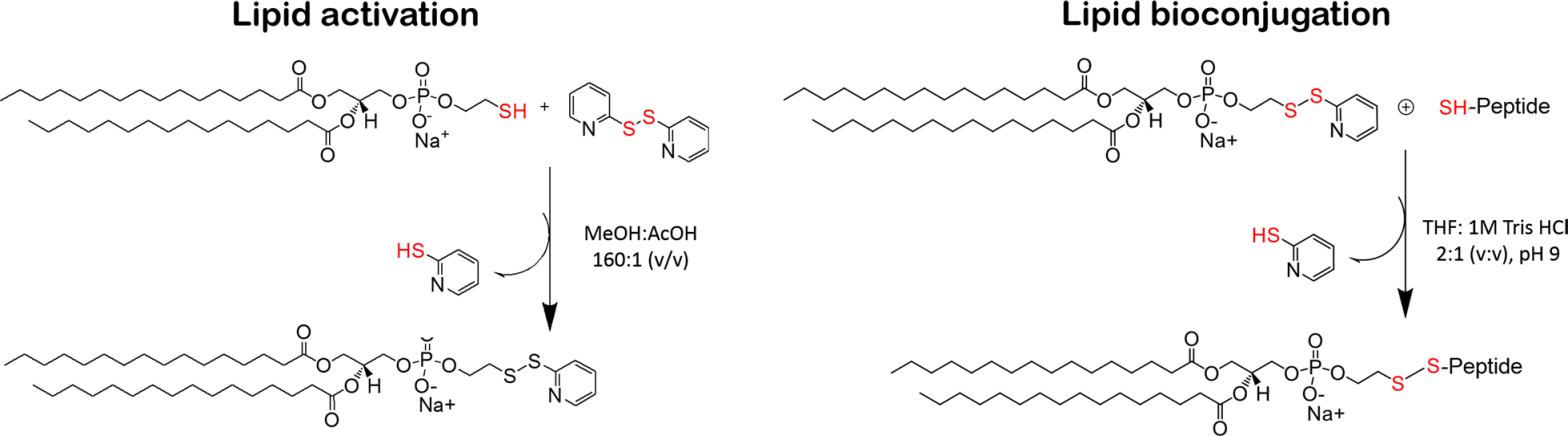
Two-step lipid–peptide conjugation through pyridyl disulfide reaction chemistry. Lipid activation (Step 1, left) DPTE lipid is activated with mercaptopyridine to avoid non-specific unwanted reaction products, i.e. the formation of the symmetric DPTE disulfide. Lipid bioconjugation (Step 2, right): the activated DPTE react with the sulfhydryl-group of the peptide of interest

The cysteine-containing tbFGF and a variant of the GALA peptide (GALA-Cys, carrying the cysteine residue at the carboxy terminal end) were conjugated to the thiol-reactive lipid 1,2-dipalmitoyl-sn-glycero-3-phospho-thio-ethanol (DPTE) accomplished with two pyridyl disulfide exchange reactions (Fig. 1). A first disulfide exchange occurs between 2-2-pyridyl disulfide (DPDS) and the thiol group of DPTE in acidic conditions (see “Methods”). After purification of the activated DPTE (aDPTE), the disulfide bridge formed by DPTE and 2-mercaptopyridine is substituted by DPTE and the cysteine-containing peptide at molar ratio of 1:2 (DPTE:peptide-SH) (see “Methods”). Although the optimal pH for disulfide exchange ranges from 4 to 5, the second disulfide exchange was performed at alkaline pH 9, above the pKa of cysteine residues (Additional file 1: Figure S1), to force the cysteine residue of the peptides to react. Furthermore, the reaction was performed in a mixture of tetrahydrofuran (THF) and 1 M Tris HCl pH 9 (2:1; vol:vol) under stirring conditions for 48 h at 20 °C in the dark. This solvent mixture ensures the solubility of both the hydrophobic lipids and the charged peptides in a buffered medium. The course and progress of this reaction can be measured spectrophotometrically (A_max_ = 363 nm) monitoring the release of the byproduct pyridine-2-thione [32]. The disulfide exchange takes place within the first 10 min where the reaction kinetic reaches a pseudo-plateau indicating a slower reaction rate (Additional file 1: Figure S2A). The reaction mixture was left up to 48 h in the dark to fully complete the reaction. After purification of the DPTE-peptide, the reaction intermediates and final products were dissolved in deuterated chloroform and characterized by ^1^H NMR spectroscopy (see Additional file 1: Figure S2B and “Methods” for details). Finally, the lipid to protein ratio of the lipo-peptide conjugation was determined by Rouser [33] and Lowry [34] assays respectively (see “Methods” for details) giving a 100% and 95% conjugation efficiency for the DPTE-tbFGF and DPTE-GALA respectively.

### POPC vesicles containing DPTE-tbFGF and DPTE-GALA

**Fig. 2:**
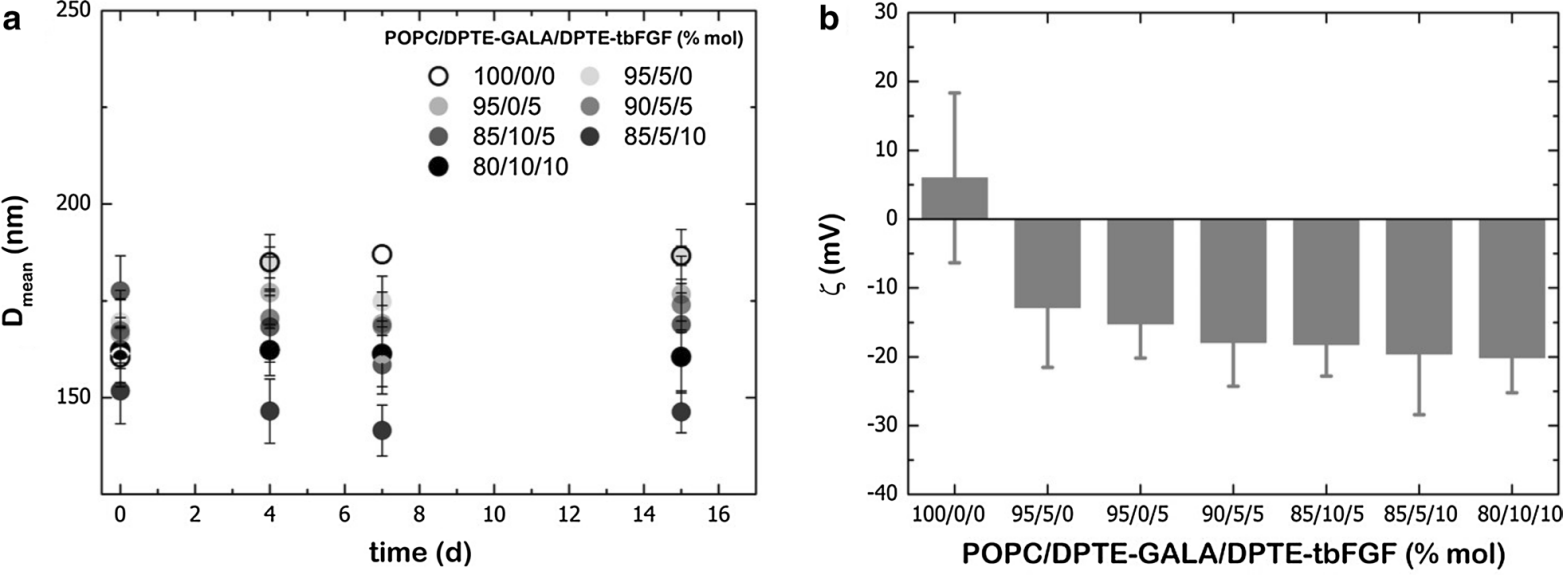
**a** Hydrodynamic diameter of POPC/DPTE-GALA/DPTE-tbFGF liposomes at different lipo-peptide molar ratios. **b** Zeta potential of POPC/DPTE-GALA/DPTE-tbFGF liposomes at different lipo-peptide molar ratios. Measurements are are representative of seven repeated experiments

To characterize the lipid concentration, the lipopeptide molar fraction, the size and the stability of liposomes, different molar ratios of DPTE-tbFGF and/or DPTE-GALA were incorporated into pure POPC vesicles. As some lipid loss might occur during extrusion [35], the lipid concentration was quantified before and after extrusion down to the size of 0.1 µm. The phosphorous analysis [33] did not detect significant loss of lipids during liposome preparation, as shown in Additional file 1: Table S1. Similarly, the molar fraction of lipopeptides was not altered during extrusion (Additional file 1: Table S1). The results obtained from the size characterization are shown in Fig. 2a. The light scattering results show that the incorporation of DPTE-tbFGF and DPTE-GALA in POPC liposomes does not affect substantially the size of the liposomes. All formulations had mean diameters around 150–200 nm immediately after extrusion and retained this size for several days. This indicates that no fusogenic nor aggregating activity of peptides is occurring at pH 7.4. However, the surface charge of POPC liposomes (~ 0 mV) is altered upon incorporation of the negatively charged peptides and a decrease in the ζ-potential is evidenced (≤ 20 mV) (Fig. 2b).

### Cell viability upon liposomal incubation with different molar ratios of DPTE-tbFGF and DPTE-GALA

**Fig. 3:**
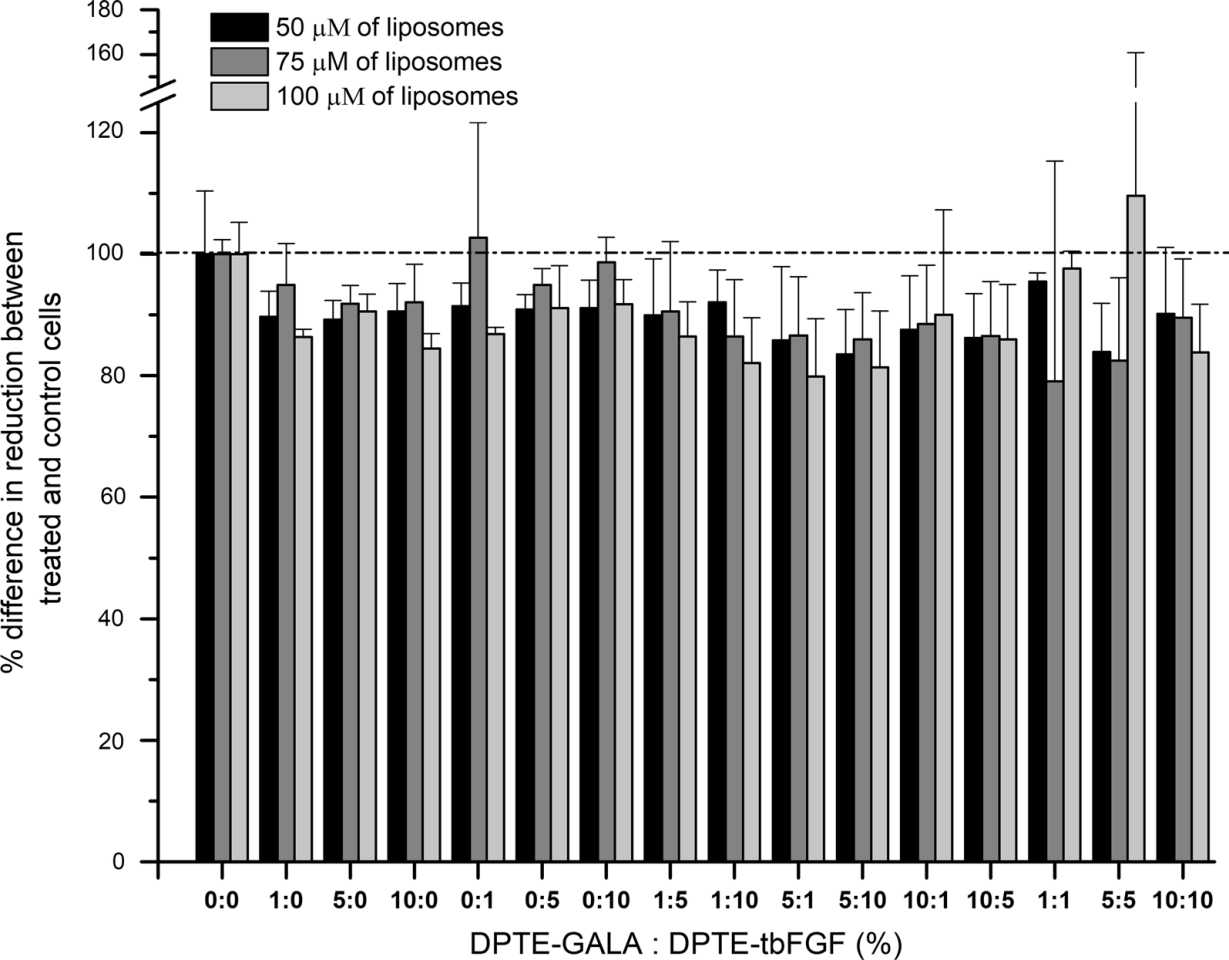
Cellular viability of mouse embryonic fibroblasts exposed to POPC liposomes decorated with DPTE-tbFGF and DPTE-GALA. MEFs were exposed to 50, 75 or 100 μM of DPTE-tbFGF and DPTE-GALA decorated POPC liposomes for 24 h at 37 °C and cell viability is assessed with the Alamar Blue reagent. The % mol ratios of DPTE-tbFGF and DPTE-GALA on the POPC liposomes are indicated in the figure (see main text for details)

We use the alamarBlue cell viability assay to assess the cell viability of mouse embryonic fibroblasts (MEFs) exposed to POPC liposomes at 50, 75 and 100 μM and decorated with different molar ratios of the lipid–peptide conjugates (DPTE-tbFGF and DPTE-GALA). Figure 3 shows the cell viability results for different molar ratios of the DPTE-peptide conjugates at 50, 75 and 100 μM liposome concentration. None of the used lipid compositions or liposomal concentrations explored showed a significant effect on cell viability (*p* value < 0.05). In general, the liposomes do not induce a significant cell death (> than 20% in extreme cases) nor do they stimulate the proliferation of MEFs. Overall, both DPTE-peptide conjugates are biocompatible, not harmful for cell viability, and thus safe to use for ex vivo MEF cultures. For following experiments with cultured MEFs we fixed the liposome concentration at 100 μM.

### Liposomal uptake of tbFGF-coated liposomes by cultured MEFs

**Fig. 4:**
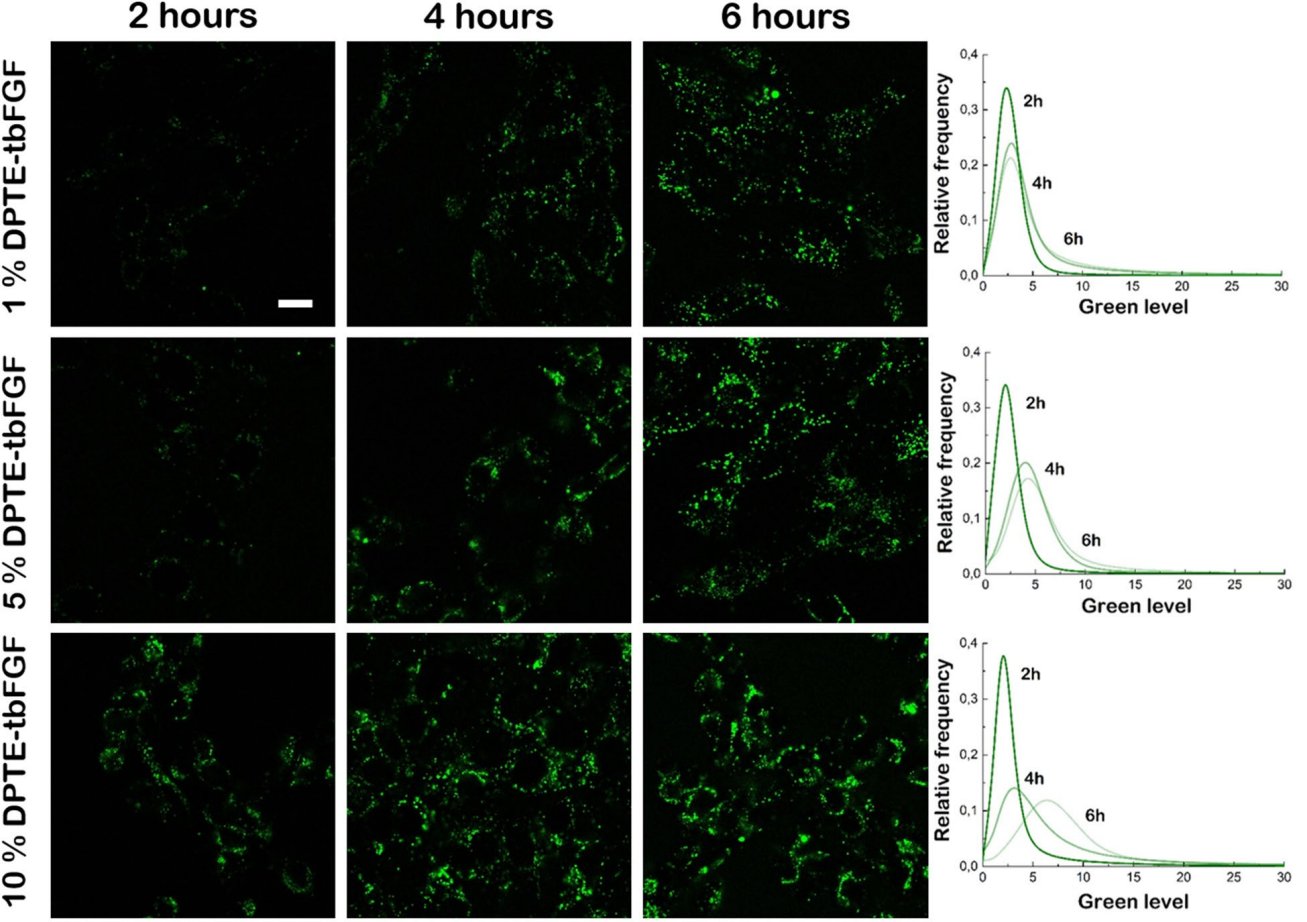
Confocal microscopy images of peptide-decorated POPC liposome uptake by mouse embryonic fibroblasts. MEFs exposed for 2, 4 and 6 h to 100 µM of calcein-loaded DPTE-tbFGF decorated POPC liposomes. The calcein fluorescence was imaged in the green channel with a bandpass of 515 to 555 nm (see main text for details). Scale bar is 10 μm

We first tested the increased uptake of tbFGF-coated POPC liposomes in cultured MEFs. MEFs were incubated with POPC liposomes presenting 0, 1, 5 and 10% molar of the targeting DPTE-tbFGF to evaluate the optimal liposome formulation for a specific uptake mediated by the tbFGF. To visualize and trace liposomes inside MEFs by means of confocal fluorescence microscopy, calcein at a final concentration of 100 mM was encapsulated in the lumen of liposomes during liposome preparation (see “Methods”). MEFs were imaged at 2, 4 and 6 h after liposome incubation. After 2 h of incubation (early uptake) liposome uptake can be observed for liposomes carrying 10% of DPTE-tbFGF. A low green fluorescence signal was also observed in cells treated with lower DPTE-tbFGF concentrations (1% or 5%) (Fig. 4, left column). After 4 h of incubation, liposome uptake is observed for all formulations used (Fig. 4, central column). At 6 h, the green level is increased for the lipid formulation carrying 10% of DPTE-tbFGF showing a high liposomal uptake. At this time point MEFs incubated with liposomes carrying 1% or 5% of DPTE-tbFGF reached comparable fluorescence intensity signals (Fig. 4, right column). For all tested conditions, control cells incubated with non-decorated bare POPC liposomes show very little, non-directed vesicle uptake, background in time (data not shown). We conclude that the presence of the DPTE-tbFGF in our liposome formulation improves the specific liposome uptake of MEFs.

### Endosomal escape of tbFGF- and GALA-coated liposomes

**Fig. 5:**
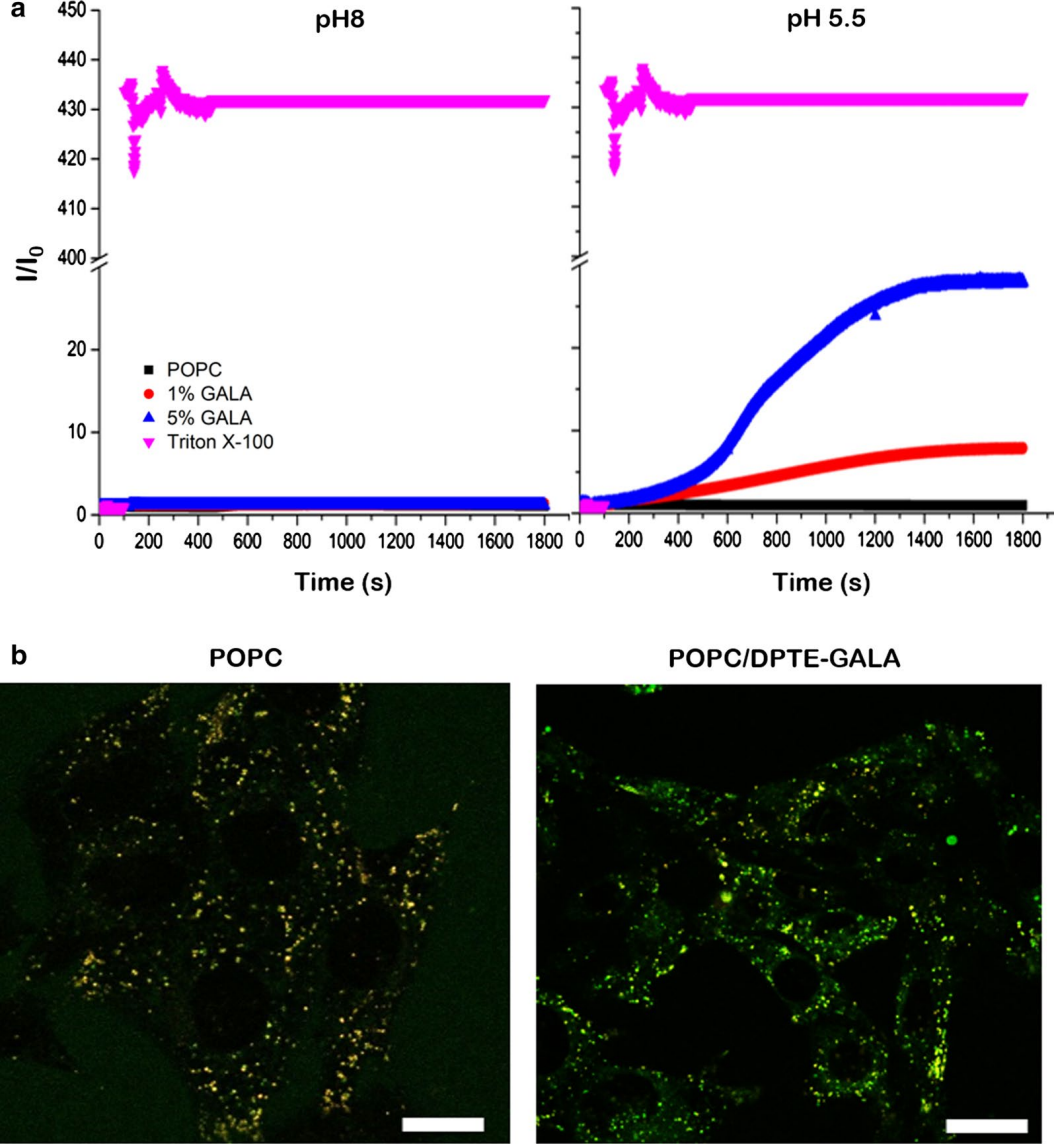
Liposome fusion assay of GALA-decorated POPC liposomes. **a** Fusion assay of GALA-decorated POPC liposomes at pH 8 and pH 5.5. The fusion was monitored through the change of the fluorescence intensity form encapsulated calcein at a self-quenching concentration of 100 mM. Total calcein release was monitored by the addition of Triton X-100 and non-decorated POPC liposomes were used as control. **b** Confocal microscopy images of MEFs exposed for 6 h to 100 µM of calcein-loaded 10% of DPTE-GALA decorated POPC liposomes at 37 °C stained with Lysotraker™ RED. The Lysotraker fluorescence was imaged in the red channel. Scale bars are 10 μm

To evaluate the fusogenic activity of DPTE-GALA conjugate, we first performed classical fluorescence liposome fusion assays with GALA-coated liposomes (1 and 5 molar ratios) carrying calcein at a self-quenching concentration of 100 mM (see “Methods”). The GALA-coated liposomes were mixed with bare and non-fluorescent liposomes. Liposome fusion leads to a dilution of lumenal calcein concentration and to an increase of the calcein fluorescence signal. Signal increase is proportional to the liposome fusion activity. At pH 5.5 an increase in fluorescence signal of GALA-coated liposomes was observed indicating liposome fusion (Fig. 5a). The velocity of the observed fusion events depends on the molar amount of DPTE-GALA (1% or 5%) attached to the liposome surface. At pH 8, no calcein release was observed, neither for the presence of 1% nor 5% DPTE-GALA, whereas the addition of the detergent Triton-X100 completely solubilized the liposomes and released the total encapsulated calcein content.

We then evaluated the fusogenic activity of DPTE-GALA inside MEFs. To visualize and trace the calcein release inside MEFs with confocal fluorescence microscopy, the cells were incubated with Lysotracker™ Red and then incubated with 100 μM POPC liposomes filled with calcein at 100 mM and decorated with 10% mol DPTE-GALA. After 12 h, the liposome uptake is observed as the fluorescence intensity of both lysosomal and calcein channels partially colocalize. We also observe calcein fluorescence in the cytoplasm of MEFs suggesting the endosomal escape of GALA-coated liposomes from the endosomal system. For comparison, bare POPC liposomes only displayed colocalization of the green and the red fluorescence signals of the liposomes trapped in the endosomal system of the cell. We conclude that the liposome uptake in MEFs is produced via the endosomal pathway and the calcein content is released by the action of the GALA lipopeptide (Fig. 5b).

### Efficient ATP delivery into MEFs

ATP is the biochemical energy of the cell and is required as many processes in cells are driven, either directly or indirectly, by the hydrolysis of ATP. Alteration of ATP biogenesis may cause a variety of severe mitochondrial disorders [36]. Classical pharmacological approaches that address the increase of the cellular levels of ATP are based on the stimulation of the de novo synthesis of ATP in situ [37] as the intravenous injection of ATP does not result in desired results because of the very short half-life of free blood circulating ATP [38]. Here, we test direct administration of ATP charged POPC liposomes [39] decorated with 10% mol DPTE-tbFGF and 10% mol DPTE-GALA.

**Fig. 6:**
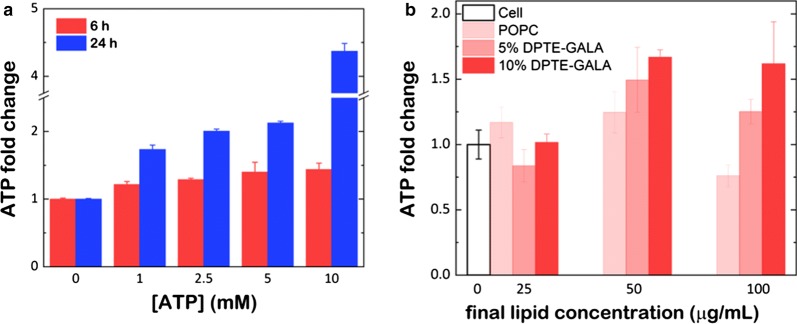
ATP delivery into mouse embryonic fibroblasts. The change of the cellular ATP concentrations in MEFs upon exposure to **a** free ATP or **b** DPTE-tbFGF- and DPTE-GALA containing POPC liposomes with encapsulated ATP. In this case, MEFs were incubated for 6 h at 37 °C and the cellular ATP levels were measured after 12 h with Luciferin/Luciferase based assay (see main text for details)

MEFs were first tested for the uptake of free ATP and incubated with increasing amounts of ATP (1, 2.5, 5 and 10 mM) and harvested after 6 and 24 h (see “Methods”). The ATP delivery in cultured MEFs was quantified with Luciferin/Luciferase based bioluminescence assay. Compared to untreated control cells, the results show a 20–40% increase of the cellular ATP level after 6 h of ATP incubation depending on the amount of ATP added to the cell culture (Fig. 6a). At 24 h, the incubation resulted in a cellular ATP level (up to fivefold increase for 10 mM ATP), but the cell viability of these cells was severely compromised suggesting that high extracellular ATP concentrations induce cytotoxic side effects including the compromising of the cell integrity (Additional file 1: Figure S3).

After the optimization of the minimal concentration of ATP required its encapsulation into POPC liposomes carrying 10% mol of DPTE-tbFGF and 10% mol of DPTE-GALA (Additional file 1: Figure S4), MEFs were incubated for 6 h with POPC liposomes containing 10% mol of DPTE-tbFGF and increasing amounts of DPTE-GALA (0, 5 and 10% mol) and loaded with 50 mM ATP. After 6 h of incubation, the liposomes were washed with fresh DMEM media and the intracellular ATP levels were quantified after 12 h. Our data show that the intracellular delivery of ATP is concentration-dependent on DPTE-GALA (Fig. 6b). Again, the formulation of POPC liposomes carrying 10% mol of DPTE-tbFGF and 10% mol of DPTE-GALA shows an improved uptake of the ATP into the cells, reaching a maximum change of ≈ 1.5-fold compared with untreated control cells.

### Protein encapsulation and release into MEFs

**Fig. 7:**
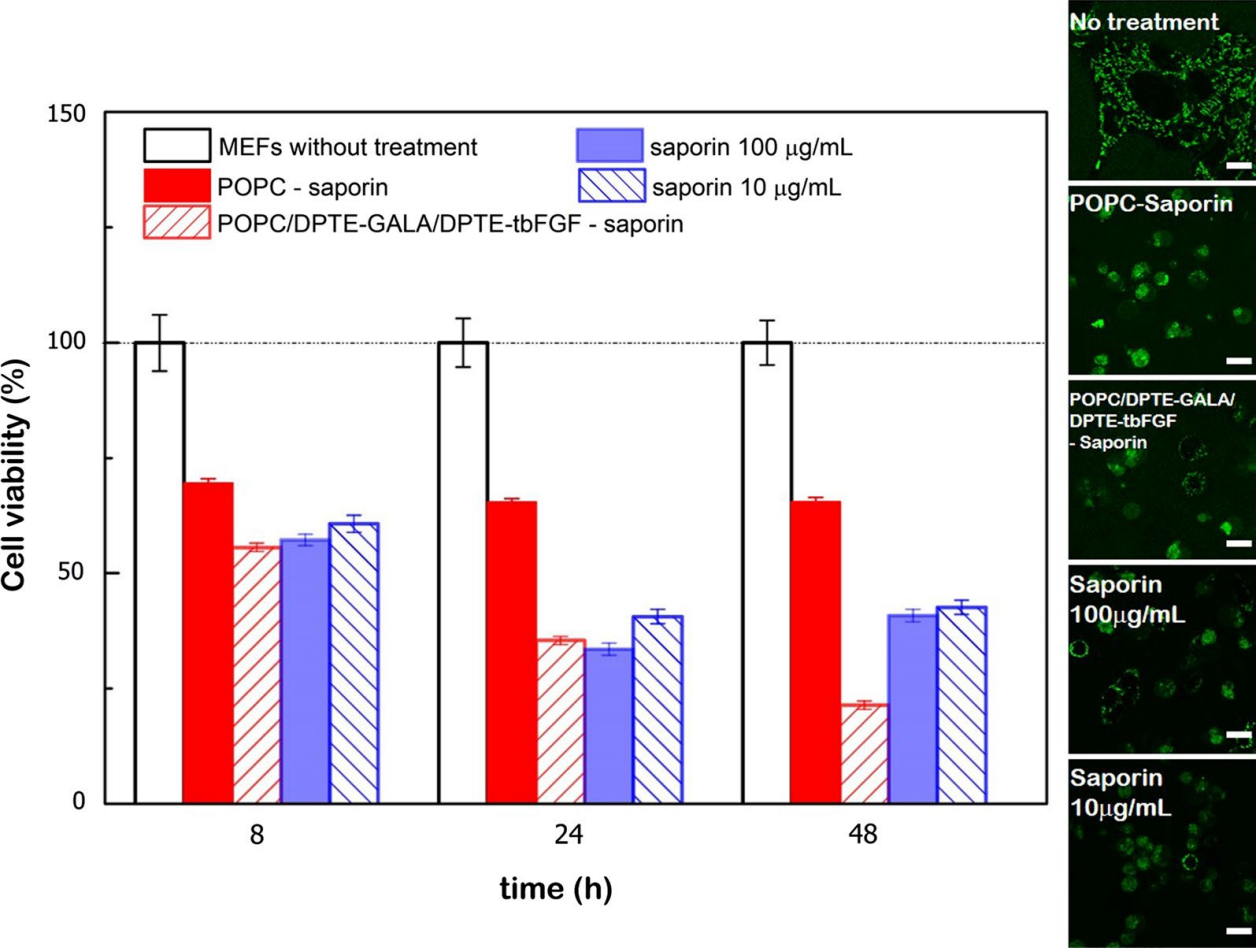
Saporin toxin delivery into mouse embryonic fibroblasts. Cellular viability of MEFs as a function of time upon saporin incubation extracellularly delivered into cells or administered with liposomes decorated with DPTE-tbFGF and DPTE-GALA. (Inset) Confocal microscopy images of MEFs exposed for 24 h at 37 °C to saporin (10 and 100 μg/ml) and DPTE-tbFGF- and DPTE-GALA containing POPC liposomes with encapsulated saporin (nominal concentration 75 μg/ml). The Rho123 fluorescence was imaged in the green channel. Scale bars are 10 μm

To unambiguously demonstrate the delivery efficiency of tbFGF- and GALA-decorated liposomes, cell viability was verified upon incubation with liposomes encapsulating the potent 30 kDa saporin-S6 toxin (saporin). Saporin enzymatically inactivates the ribosomes leading to a block in de novo protein synthesis, resulting in the cell death [40, 41]. When the MEFs were exposed to 10 or 100 µg/ml of non-encapulated saporin, ∼ 50 to 60% of a cell death was observed after 8 and 48 h of incubation respectively. However, 30 to 35% of cell death was produced when the toxin was administered in “bared” POPC liposomes. Remarkably, the presence of 10% mol DPTE-GALA and DPTE-tbFGF progressively decreased the cell viability and killed 75% of the cells after 48 h of incubation (Fig. 7).

The cytosolic delivery of saporin was further confirmed by scanning confocal fluorescence microscopy. MEFs were labelled with the mitochondrial marker Rho123. Over time, the normal mitochondrial network of MEFs remained unaltered in the absence of treatment. In contrast, when non-encapsulated saporin was extracellularly supplemented to the medium and delivered into cells or administered through liposomes, a significant cell shrinkage and a change in mitochondrial morphology, indicating cell death, was observed (inset in Fig. 7). These results collectively demonstrate that DPTE-based lipopeptides allows the delivery of proteins without the loss of functionality into the cytosol of eukaryotic cell.

## Discussion

In drug delivery, the development of experimental techniques for the effective release of liposomal contents through endosomal and lysosomal membranes is key to prevent their degradation through the endosomal maturation pathway before reaching cytosolic moiety of the cell. Our finding, a peptide decorated liposome formulation, allows the specific cellular uptake and the escape of the luminal content from the endosomal system for efficient drug delivery.

In this work we used two peptides, the tbFGF and GALA to control the efficient cellular uptake and endosomal escape of our POPC liposome vector respectively. The tbFGF peptide is a truncated version of the fibroblast growth factor (FGF) that plays an important role in tumor growth and angiogenesis [42–44]. This peptide binds to the bFGF receptor but does not stimulate cell proliferation [45]. Endocytosis is the major pathway for the tbFGFp mediated cellular uptake of liposomes for cell-to-cell communication and their use in drug delivery [22, 46–51]. The GALA peptide was designed to mimic viral fusion protein sequences that interact with cell membranes to mediate the escape of the viral genes from acidic endosomes [28]. The application of the GALA peptides is well studied and used in gene transfection [52–57] and the cytosolic delivery of peptides and proteins [47]. In more detail, we conjugated DPTE to the cysteine peptides tbFGF and GALA-Cys prior to lipid vesicle preparation. The lipopeptide synthesis method (Fig. 1), based in the pyridyl disulfide reaction chemistry [21], is characterized by high yields and very easy purification procedures and differs from previously reported cysteine conjugation methods [19], which result in succinimide bonds that can be hydrolyzed spontaneously and give thus in very low coupling efficiencies. Our approach relies on the formation of a pre-conjugated nano-sized peptide-lipid system prior to their incorporation into the liposomes; whereas most of previous works dealt with some thiol-reactive polymers [58–60] or pre-formed nano-objects [21, 61, 62] that were designed to react with the cysteine residue of the targeted peptide or protein. Despite the high versatility of the thiopyridyl group, its reactivity depends on three main parameters: i.e. the size of the object bearing the disulfide function, the size of the molecule bearing the thiol group and the pH value [63]. Also, the steric hindrance of the thiol group has been found to be crucial as the kinetics of the thiol-disulfide exchange is drastically affected when a small thiol-bearing substrate was replaced by a larger one [63]. It has been also reported that the conjugation of liposomes with proteins might lead to liposome aggregation and/or a mixture of labeled and unlabeled liposomes that was difficult to resolve [64]. Our approach is thus suitable to functionalize lipids prior to liposome formation. To our knowledge, only few reports [65] follow the same strategy as ours. The advantage of our method resides on the easy modulation and control of the amount of lipopeptides to be incorporated into the liposomes.

Moreover, DPTE is commercially available and provides a stable membrane anchoring of peptides to the lipid bilayer in comparison with single fatty acid chain derivatives. The resulting lipopeptides are soluble in organic solvents such as methanol or chloroform and can be incorporated into liposomes in the same manner as common phospholipids. This allows the controlled surface decoration and the controlled mixing of different peptides on the liposome surface without loss of peptide functionally and ensures the peptide anchoring to the lipid bilayer without impairing the stability and size of liposomes (Fig. 2a). In particular, we demonstrate the pH-dependent fusogenic properties of liposomes containing DPTE-GALA in a physiologically relevant pH-range (Fig. 5a). The liposomes can fuse with stable target liposomes with a size that mimics the endosomal vesicles in cells. The lipopeptide DPTE-GALA presents similar characteristics of GALA lipopeptides previously described in the literature, where two myristoyl chains were attached to the end of GALA through 1,2-diamino propanoic acid, yielding the lipopeptide DMDGALA [66]. The pH-dependent fusion of DPTE-GALA containing liposomes allows them to be used for drug delivery applications. To this end, the effects of adding the targeting lipopeptide DPTE-tbFGF to the liposome surface was investigated. The combined addition of different DPTE-based lipopeptides (up to 20% mol of liposome composition) might compromise cell viability. However, we have found that the action of both DPTE-peptides are biocompatible and does not produce cytotoxicity (Fig. 3).

To establish the optimal formulation for efficient cellular uptake and drug release, MEFs were exposed to POPC liposomes decorated with 0 to 10% of DPTE-tbFGF and/or DPTE-GALA and incubated for 6 h at 37 °C. Confocal microscopy imaging shows the effective cellular uptake of calcein-loaded liposomes and the cytosolic release of its content. An enhanced uptake is observed with increasing amounts of DPTE-tbFGF (Fig. 4) and no significant cytosolic spread of calcein was observed in the absence of DPTE-GALA (Fig. 5). These observations strongly suggest the involvement of endocytosis in the cellular uptake. Additional experiments inhibiting the cellular uptake are required to quantitatively ascertain the importance of endosomal pathway and ulterior acidification in this targeting system. At low temperature (4 °C) the energy-driven processes including endocytosis are suppressed and the presence of ammonium chloride, prevents endosomal acidification [67]. Nonetheless, liposomes attaching GALA are proficient for membrane disruption at low pH in living cells. This was previously shown in vitro for GALA attached to a flat gold surface [68]. In our case, an increase in DPTE-GALA in the presence of 10% mol DPTE-tbFGF, led to a faster cytosolic delivery of calcein, however we did not quantify the efficiency of the delivery. No significant cytotoxicity was observed during these incubations even when both, DPTE-tbFGF and DPTE-GALA were present up to 10% mol. However, a larger excess of both DPTE-GALA and DPTE-tbFGF does not necessarily yield to an increased and more efficient cytosolic drug release. The excess of DPTE-GALA might even increase the negative charges on the liposomal surface (Fig. 2b) and interfere with specific DPTE-tbFGF mediated cellular uptake [57]. We did not investigate the fate of the liposome components after drug delivery, but expect them to be degraded and completely cleared from the cells [69].

For a supplementary piece of evidence of quantitative delivery, we loaded liposomes with ATP and monitor the cellular ATP levels. After incubation with peptide-coated liposomes, we were able to increase the ATP levels in MEFs (Fig. 6). The addition of extracellular (non-capsulated) ATP to cell cultures is known to be toxic at high concentrations [70], thus encapsulation might allow efficient cytosolic delivery of ATP without the need of de novo synthesis. The presence of both tbFGF as well as the GALA is essential for the efficient cytoplasmic release of ATP. This preliminary data indicates that our formulation might be a favorable combination needed for efficient cytosolic targeting and release. However, the ATP quantification is carried out after cell lysis and therefore the endosomal contents might be released during manipulation and the quantification biased. A concluding demonstration of the delivery efficiency of tbFGF- and GALA-coated liposomes came from the encapsulation and the release of saporin toxin that blocks the de novo protein synthesis by inactivating the ribosomes in the cytosol. Our results (Fig. 7) demonstrate an enhanced cytotoxicity when the toxin was delivered through liposomes enriched in DPTE-GALA and DPTE-tbFGF as compared to controls. In contrast to a single dose of high concentrated saporin, the combined action of lipopetides might mediate a progressive release of the saporin toxin producing a gradual enhanced cytotoxicity that might counterbalance the gradual cell recovery eventually leading to a higher cell death (Fig. 7).

Overall, the optimal combination of POPC liposomes enriched with DPTE-GALA and DPTE-tbFGF (molar ratios of 8:1:1) was important to attain an effective balance of internalization and cytosolic release and was found to be biocompatible in mouse embryonic fibroblasts. The success of our liposome formulation can be attributed to the presence of DPTE, a thiol-containing lipid that can efficiently be incorporated into biological membranes [71, 72].

## Conclusion

We used a straightforward methodology for the formulation of peptide-decorated liposomes to ensure the efficient enhanced cellular uptake and cytosolic release of encapsulated cargo. The targeted peptide tbFGF and the pH-sensitive fusogenic peptide GALA were conjugated via cysteine residue to the thiol-containing phospholipid DPTE prior to vesicle preparation. Although further studies are needed for more advanced and fine-tuned control of the cellular uptake and cytosolic release inside live cells, our formulation strategy will greatly contribute to the application of drug delivery to assess therapeutic remedies to cells with pathological phenotypes.

## Methods

### Lipids, fluorescent probes, peptides and toxin

1-Palmitoyl-2-oleoyl-*sn*-glycero-3-phosphocholine (POPC), and 1,2-dipalmitoyl-sn-glycero-3-phosphothioethanol (DPTE) were supplied by Avanti Polar Lipids. The peptides NH2-KRTGQYKLC-COOH and NH2-WEAA-LAEA-LAEA-LAE-H-LAEA-LAEA-LEALAAC-COOH were synthesized by GenScript (Piscataway, NJ USA) and used without any further purification step. Lysotraker™ RED (L7528) was purchased form Thermofisher. Saporin (S9896), calcein (21030, Fluka) and Rhodamine 123 (R8004) were purchased from Sigma Aldrich.

### Conjugation of the cysteine-containing peptides with the lipid 1,2-dipalmitoyl-sn-glycero-3-phospho-thio-ethanol

The conjugated lipopeptides were synthetized in two steps. The first step consists on the activation of the thiol group of the lipid 1,2-dipalmitoyl-sn-glycero-3-phosphothioethanol (DPTE; Avanti Lipids) and the second step is the conjugation of the reactive dissymmetric disulfide DPTE (aDPTE) with the cysteine residue of the tbFGF and GALA-Cys peptides (GenScript, Piscataway, NJ USA). In more detail, 200 mg (275 μmol) of DPTE and 120 mg (550 μmol) of 2,2′-dipyridyldisulfide (DPDS) were first dissolved in methanol:acetic acid (MeOH:AcOH 160:1, v/v), in a final volume of 4 ml and incubated under stirring for 48 h at RT in the dark. For the second reaction, 5 mg (6 μmol) of aDPTE was incubated with 40 mg (40 μmol) of tbFGF or 120 mg (40 μmol) of GALA-Cys (1:7 mol:mol aDPTE:peptide ratio) in a mixture of tetrahydrofuran (THF) and 1 M Tris HCl pH 9 (2:1; vol:vol) in a final volume of 3 ml and stirred for 48 h at 20 °C in the dark. The release of mercaptopyridine in both reactions was monitored spectrophotometrically (Genesis 10 spectrophotometer; Fisher Scientific) at 362 nm. The absorption spectra of the reaction mixture were recorded during incubation at 20 °C in disposable cuvettes with an optical path of 1 cm from 300 nm to 500 nm with a spectral resolution of 1.0 nm and a scan rate of 200 nm/min. The reaction takes place within the first 10 min where mercaptopyridine is released very fast, but is left up to 48 h to ensure complete reaction of the substrates. During the synthesis, samples were analyzed by thin layer chromatography by comparing the R_*f*_ values of reaction products with protein and lipid standards.

### Thin layer chromatography

Preparative silica TLC [73] was used to remove mercaptopyridine from conjugation reaction using acetone as the mobile phase. The mercaptopyridine runs in the acetone front leaving both, the aDPTE and the DPTE-peptide, behind on the silica plate. Both the aDPTE and DPTE-peptide were scraped from the preparative TLC plate (Analtech, USA) and dissolved in 30 ml of chloroform and applied on a silica column for further purification.

### Column preparation and elution

Silica Gel (Sigma-Aldrich), suspended in chloroform, was packed to a height of 30 cm column. Applying slight air pressure during packing resulted in a uniform distribution of adsorbent, which was supported and covered by small glass wool plugs. The scraped lipid-silica mixture was suspended in chloroform and applied to the prepacked column and washed with three column volumes of chloroform:methanol mixture (CHCl_3_:MeOH; 9:1: v/v) to elute the lipid mixture into the column. The DPTE and DPTE-peptide will dissolve in the mobile phase whereas the silica will remain packed on the prepacked solid silica phase. Elution was carried out with three column volumes of CHCl_3_:MeOH (13:5; v/v) at room temperature under slight air pressure. The eluate was collected in 30 ml fractions and analyzed by TLC to detect the presence of DPTE or DPTE-peptide. Lipids were visualized with the molybdic oxide–molybdenum “Zinzadze” phosphorus staining reagent [74]. The fractions containing the aDPTE or DPTE-peptide were pooled individually, the solvents evaporated under nitrogen and the residual lipids or conjugates stored at − 20 °C until further use. The lipid and protein content of the obtained products were determined according to Rouser [33] and Lowry [34] respectively to calculate the efficiency of synthesis. The synthesized products (aDPTE and DPTE-peptide) were characterized by 1 H NMR spectroscopy (NMR CAI; Universidad Complutense Madrid).

### Preparation of liposomes

Liposomes were prepared according to the standard thin-film hydration method [75]. For the different formulations used here, the lipid and DPTE-peptide containing chloroform solutions were mixed and dried using a vacuum concentrator (Eppendorf). Dried films were then hydrated with 500 µl of in Ca^2+^ and Mg^2+^ free PBS (the final lipid concentration was 1 mg/ml) and vortexed for 10 min facilitating hydration of the lipids and the formation or multilamellar lipid vesicles (MLVs). After hydration the sample was extruded through a polycarbonate membrane (100 nm pore size; Avanti lipids) and exposed to MEFs grown in DMEM (see “Cell cultures”) for viability, uptake and release assays. For confocal experiments, calcein (100 mM) was added to buffer prior to rehydration step. Calcein containing MLVs were dispersed by vortexing (5 min) and sonication of the turbid suspension up to 20 min. Liposomes were washed trice with 40 mM HEPES by ultracentrifugation (Beckmann, TLA120 rotor, 100,000×*g*, 60 min) and resuspended in a final volume of 500 µl of 50 mM HEPES, 150 mM KCl, pH 8 for lipid-mixing assays or Ca^2+^ and Mg^2+^ free PBS for cell assays. For ATP and saporin delivery experiments, the lipid film was rehydrated in the presence of 0 mM (control), 10 mM, 25 mM 50 mM and 100 mM of ATP buffered in 1 M HEPES pH 7.2 or 75 μg/ml of saporin buffered in 100 mM HEPES pH 7.2. ATP or saporin containing MLVs were dispersed by vortexing (5 min) and sonicated up to 20 min until they became transparent. Liposomes were washed trice with 1 M HEPES pH 7.2 or in 100 mM HEPES pH 7.2 by ultracentrifugation (Beckmann, TLA120 rotor, 100,000×*g*, 60 min) and resuspended in a final volume of 100 µl of 1 M HEPES pH 7.2 or in 100 mM HEPES pH 7.2 at a final lipid concentration of 1 mg/ml. The final concentration upon incubation with MEFs was achieved after further dilution in DMEM media.

#### Chemical analysis, DLS and zeta potential of liposomes

The lipid and lipopeptide content of liposomes before and after extrusion were also determined according to Rouser [33] and Lowry [34]. The diameters and the zeta-potential of the liposomes were measured as a function of the molar ratio of lipopeptides by dynamic light scattering (DLS) and electrophoretic mobility measurements in a 90 Plus Particle Analyzer (Brookhaven Instruments).

### Liposomes fusion assay

15 mM POPC/DPTE-GALA liposomes carrying increasing amount of DPTE-GALA (1, 5% mol) loaded with calcein at self-quenching concentration of 100 mM were incubated with empty 15 mM POPC liposomes (1:9 vol:vol) and titrated with 0.1 M HCl to drop the external pH from 8 to 5.5. The fusion assay is based on the dilution of the lumenal calcein concentration that leads to an increase of the fluorescence signal. The fluorescence signal was monitored spectroscopically on an AMINCO-Bowman Series 2 (AB2) Spectrofluorometer (emission wavelength of 520 nm upon excitation at 495 nm and slit width of 5 nm). The maximal calcein release of the POPC-DPTE-GALA liposomes was estimated after solubilization of 1% of triton X-100 at pH 8 and 5.5.

### Cell culture

The mouse embryonic fibroblasts (MEF; purchased from ATCC) were cultured in complete DMEM (high glucose Dulbecco Modified Eagle Medium), 25 mM Glucose (Gibco) supplemented with 10% fetal bovine serum (South Africa S1300; Biowest, Nuallé, France), penicillin/streptomycin (final concentration 100 U/ml of penicillin and 100 μg/ml of streptomycin respectively) and 1% of non-essential amino acids (all Gibco). The cells were grown in a humidified incubator (Forma Steri-Cycle Themofisher; 5% CO_2_) at 37 °C and maintained, with split ratio of 1:20, at 80% of confluence in T75 flasks (Nunc).

### Cell viability assays

The cell viability of treated MEFs was monitored with the Alamar Blue viability assay (Serotec, Oxon, UK) [76–78] according to manufacturer’s instructions. This cell viability assay is based on a reazurin that reflects the redox state of the cell. In living cells, reazurin (7-Hydroxy-3H-phenoxazin-3-one 10-oxide) is effectively reduced due to mitochondrial metabolic activity, where NADPH or NADH reduces the blue resazurin to the red resorufin [79]. Reazurin absorption was measured at 570 nm and data was corrected according to:

1 $$\frac{{\left( {\varepsilon_{ox} } \right)\lambda_{2} {\text{A}}\lambda_{1} - \left( {\varepsilon_{ox} } \right)\lambda_{1} {\text{A}}\lambda_{2} }}{{\left( {\varepsilon_{ox} } \right)\lambda_{2} {\text{A}}^{o} \lambda_{1} - \left( {\varepsilon_{ox} } \right)\lambda_{1} {\text{A}}^{o} \lambda_{2} }} \times 100$$

with λ_1_ = 570 nm and λ_2_ = 620 nm, ε_ox_ = 80,586 L mol^−1^ cm^−1^ at 570 nm, Aλ_1_ the absorption at 570 nm and Aλ_2_ the absorption at 620 nm, the superscript º stands for the positive control well. MEFs were lifted and seeded in 96-well plates at a density of 3 × 10^3^ cells/cm^2^. After 24 h of incubation at 37 °C with 5% CO_2_ and 95% humidity in the cell incubator to allow cell attachment, 10 μl of the Alamar Blue reagent is added to each well of the 96-well plate and incubated for additional 2 h at 37 °C with 5% CO_2_ and 95% humidity in the cell incubator. After incubation, absorption of individual plate was measured at 570 nm and 620 nm in a Multiskan™ FC (Thermo Scientific™) plate reader. Data was analyzed with a two-way ANOVA (p-value < 0.05) relating cell death to the ratios of DPTE-GALA/DPTE-tbFGF and the total concentration of transfected liposomes (50, 75 and 100 μM) or ATP concentration.

### Confocal laser scanning microscopy

Confocal laser scanning microscopy images were taken of MEFs seeded in a four-chamber LabTeck^®^ (C6807, Sigma-Aldrich) at a density of 1 × 10^5^ cells per cm^2^ in complete DMEM and incubated for 24 h at 37 °C. Before microscopy MEFs were washed twice with HBBS and incubated in complete DMEM medium containing 100 μM of liposomes. Cells were imaged at 2, 4 and/or 6 h after liposome incubation. The LabTeck^®^ chamber was mounted on a stage of a Nikon Ti-E inverted microscope at 37 °C equipped with a Nikon C2 confocal spot microscope, Nikon Plan Apo 100× NA oil immersion objective 1.45 UV filter cubes-2E/C (excitation band 340–380 nm, emission band 435–485 nm), B-2E/C (excitation band: 465–495 nm, emission band: 515–555 nm and Y-2E/C (excitation band: 540–580 nm, emission band: 600–660 nm). Images were captured with Nikon NIS-Elements software and processed with ImageJ software package [80].

### Luciferase assay

Intracellular ATP and encapsulated ATP in the liposome were quantified using ATP Determination Kit (Thermo Fisher Scientific, USA) and performed according to manufacturer instructions. For ATP or ATP encapsulated liposome treatment, MEFs were grown on 96-well plate by seeding 1.5 × 10^4^ cells/well. After 24 h of incubation at 37 °C with 5% CO_2_ and 95% humidity in the cell incubator each well was treated with 100 µl of DMEM with appropriate concentration of ATP or liposome and measured the intracellular ATP at different time points. For quantification of ATP, the cells were washed three times with PBS to remove the unbound ATP. Thereafter 100 µl lysis buffer (0.2 M borate buffer, 0.1% Triton X100, pH 9.2) was added to each well and the plates were kept at room temperature for 10 min. The resultant lysate was further diluted with 200 µl lysis buffer then transferred to a 1.5 ml eppendorf tube. The cellular debris were removed by centrifugation at 13,500×*g* and 4 °C for 10 min and supernatant were transferred to new eppendorf tube. 10 µl of the supernatant was used for ATP determination assay. To determine the encapsulated ATP in the liposome, 50 µl liposomes were lysed using 50 µl lysis buffer as mentioned above, incubated for 10 min and then diluted three-fold by the addition of 200 µl lysis buffer. 10 µl of lysate was used to determine the ATP concentration. Luminescence assay was performed with a white 96-well plate and luminescence was measured with a microplate reader at 560 nm (BMG Labtech, Germany).

## Additional file

**Additional file 1.** Additional figures and table.

## Data Availability

All data generated or analyzed during this study are included in this published article.

## Abbreviations

1H-NMR: nuclear magnetic resonance
aDPTE: activated 1,2-dipalmitoyl-sn-glycero-3-phosphothioethanol
bFGF: basic fibroblast growth factor
tbFGF: truncated basic fibroblast growth factor
DMEM: Dulbecco’s modified Eagle Medium
DPDS: 2-2-pyridyl disulfide
DPTE: 1,2-dipalmitoyl-sn-glycero-3-phosphothioethanol
GALA peptide: 30 amino acid synthetic peptide with glutamic acid-alanine-leucine-alanine (EALA) repeat
GALA-Cys: GALA peptide with C-terminal cysteine residue
HBBS: Hank’s buffered salt solution
MEF: mouse embryonic fibroblasts
PBS: phosphate buffered saline
