# Supplementary material for: Lipid–peptide bioconjugation through pyridyl disulfide reaction chemistry and its application in cell targeting and drug delivery
Source: J Nanobiotechnology. 2019 Jun 21;17:77. doi: 10.1186/s12951-019-0509-8 (PMC6587267; doi:10.1186/s12951-019-0509-8)
Supplement: Supplementary file 1 — Additional file 1. Additional figures and table. [file 12951_2019_509_MOESM1_ESM.docx]

# Additional files

**Lipid-peptide bioconjugation through pyridyl disulfide reaction chemistry and its application in cell targeting and drug delivery**

Diego de la Fuente-Herreruela^a,b^, Ajay K. Monnappa^c^, Mónica Muñoz-Úbeda^b^, Aarón Morallón-Piña^a^, Eduardo Enciso^a^, Luis Sánchez^d^, Fabrice Giusti^e^, Paolo Natale^a,b^ and Iván López-Montero^a,b^*

a. Dto. Química Física, Universidad Complutense de Madrid. Avenida Complutense s/n 28040 Madrid, Spain

b. Instituto de Investigación Hospital Doce de Octubre (i+12). Avenida de Córdoba s/n 28041 Madrid, Spain

c. School of Life Sciences, Department of Biological Sciences, Ulsan National Institute of Science and Technology (UNIST), Ulsan 689-798, Republic of Korea.

d. Dto. Química Orgánica, Universidad Complutense de Madrid. Avenida Complutense s/n 28040 Madrid, Spain

e. Institut de Chimie Séparative de Marcoule, ICSM - UMR 5257, Site de Marcoule – Bât. 426 BP 17 171, 30207 Bagnols sur Ceze, France

[*ivanlopez@quim.ucm.es](mailto:*ivanlopez@quim.ucm.es)

**Table S1.** Recovery of the lipids and DPTE-peptides after extrusion.

| *Composition*  *POPC/DPTE-GALA/DPTE-tbFGF*  *(mol:mol:mol)* | *Phosphorous concentration* before extrusion (mM)* | *Phosphorous concentration* after extrusion (mM)* | *Lipopetide*  *molar fraction***  *before extrusion (% mol)* | *Lipopetide*  *molar fraction***  *after extrusion*  *(% mol)* |
| --- | --- | --- | --- | --- |
| 100/0/0 | *0.9 ± 0.2* | *1.0 ± 0.2* | *-* |  |
| 95/5/0 | *1.2 ± 0.1* | *1.1 ± 0.3* | *4.9 ± 0.5* | *4.8 ± 0.5* |
| 95/0/5 | *1.2 ± 0.1* | *0.9 ± 0.1* | *5.2 ± 0.3* | *4.6 ± 0.4* |
| 90/5/5 | *1.2 ± 0.3* | *1.2 ± 0.2* | *9.7 ± 1.0* | *9.8 ± 0.9* |
| 85/10/5 | *1.3 ± 0.1* | *1.1 ± 0.1* | *16.1 ± 1.8* | *15.0 ± 1.7* |
| 85/5/10 | *1.2 ± 0.3* | *1.0 ± 0.2* | *17.2 ± 1.7* | *15.2 ± 1.8* |
| 80/10/10 | *1.3 ± 0.2* | *1.1 ± 0.2* | *20.1 ± 2.0* | *19.7 ± 2.6* |

Data represent the mean (± standard deviation) of one single experiment performed in triplicate. *The phosphorous con**t**ent and ** the peptide content was determined according to Rouser and Lowry, respectively that allowed the determination of lipid to protein ratio of liposomes (See Methods for details).

**
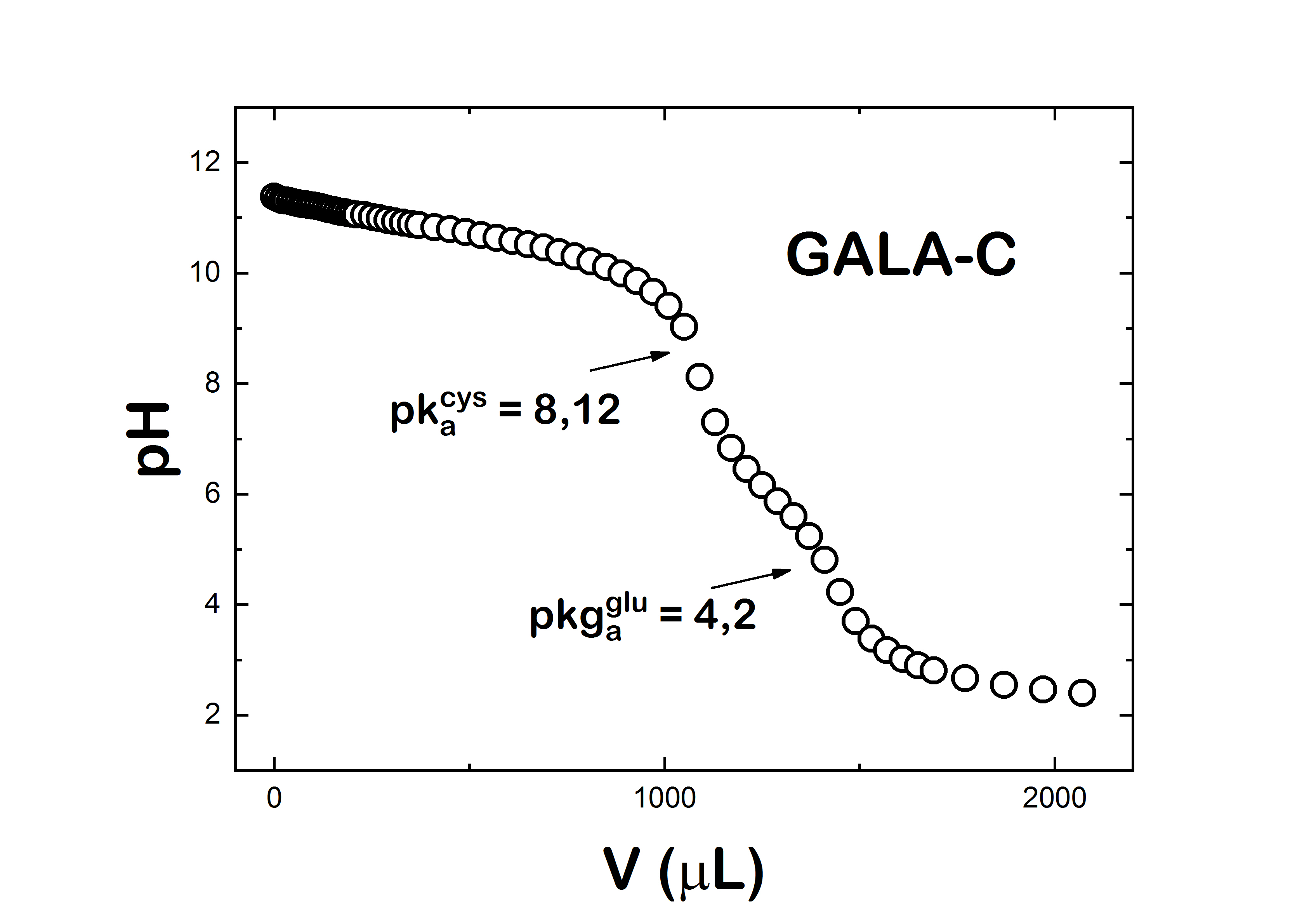
**

## Figure S1. Titration curve of the cysteine residue of the GALA-cys peptide. The GALA-cys peptide exhibits a pK_a1_ of 8.12 and a pK_a2_ of 4.2 corresponding to glutamic acid and cysteine (See Ref. Hass MA, Mulder FA. 2015. Contemporary NMR Studies of Protein Electrostatics. Annu Rev Biophys. 44: 53-75). To favor the conjugation with the activated lipid the experiment was carried out at pH 9.

**
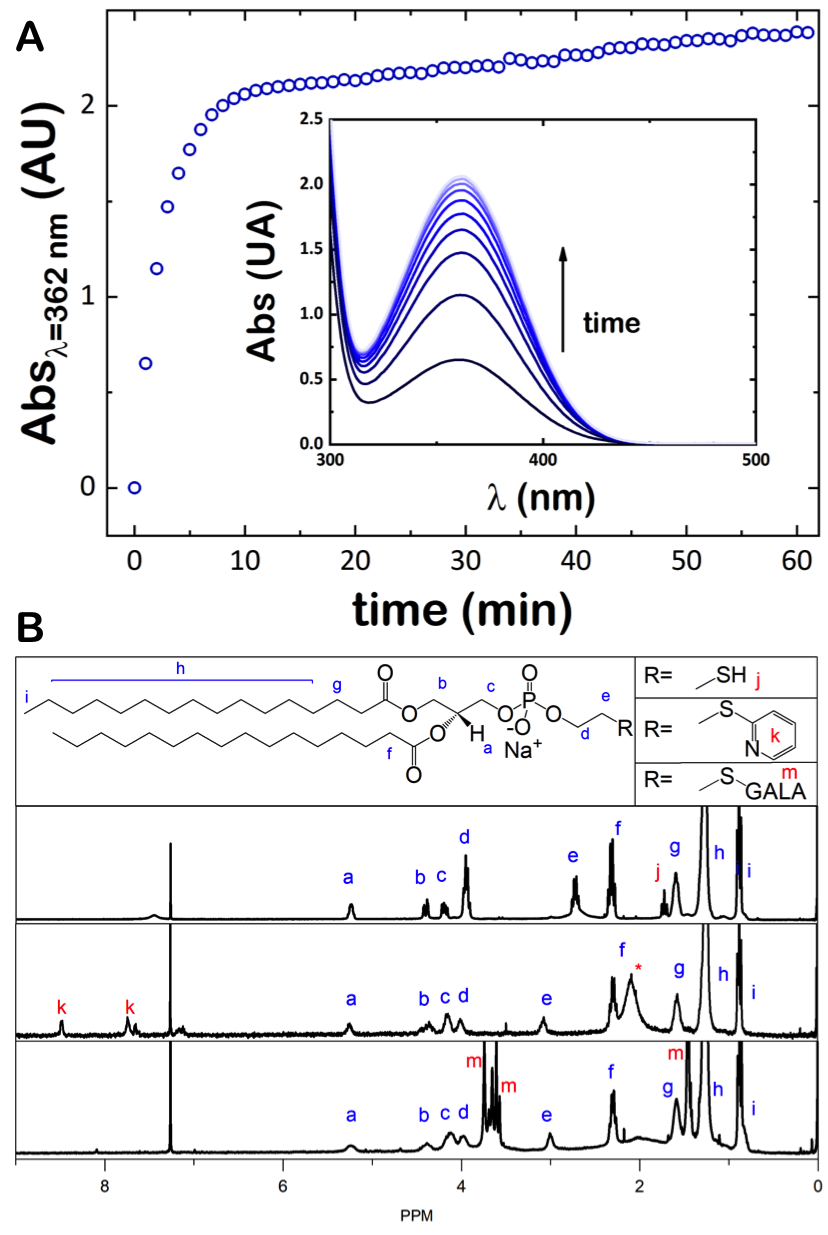
**

## Figure S2. The synthesized DPTE-GALA bioconjugate. A) Absorption spectra of mercaptopyridine release (inset) and the reaction kinetics of aDPTE:GALA (1:1; mol:mol) conjugation reaction in a mixture of tetrahydrofuran (THF) and 1M Tris HCl pH 9 (2:1; vol:vol) at 20ºC in the dark. The reaction takes place within the first 10 minutes where mercaptopyridine is released very quickly, but reaction was left up to 48 hrs to ensure complete reaction of the substrates. B) ^1^H NMR spectra in deuterated chloroform of reaction substrate DPTE (top NMR spectrum), the product aDPTE of the first reaction (central NMR spectrum) and the product DPTE-GALA of the bioconjugation (second reaction) (bottom NMR spectrum). Letters a to m correlate the obtained NMR signals with molecular structure and the asterisk mark in the central NMR spectrum indicates and non-identified peak.


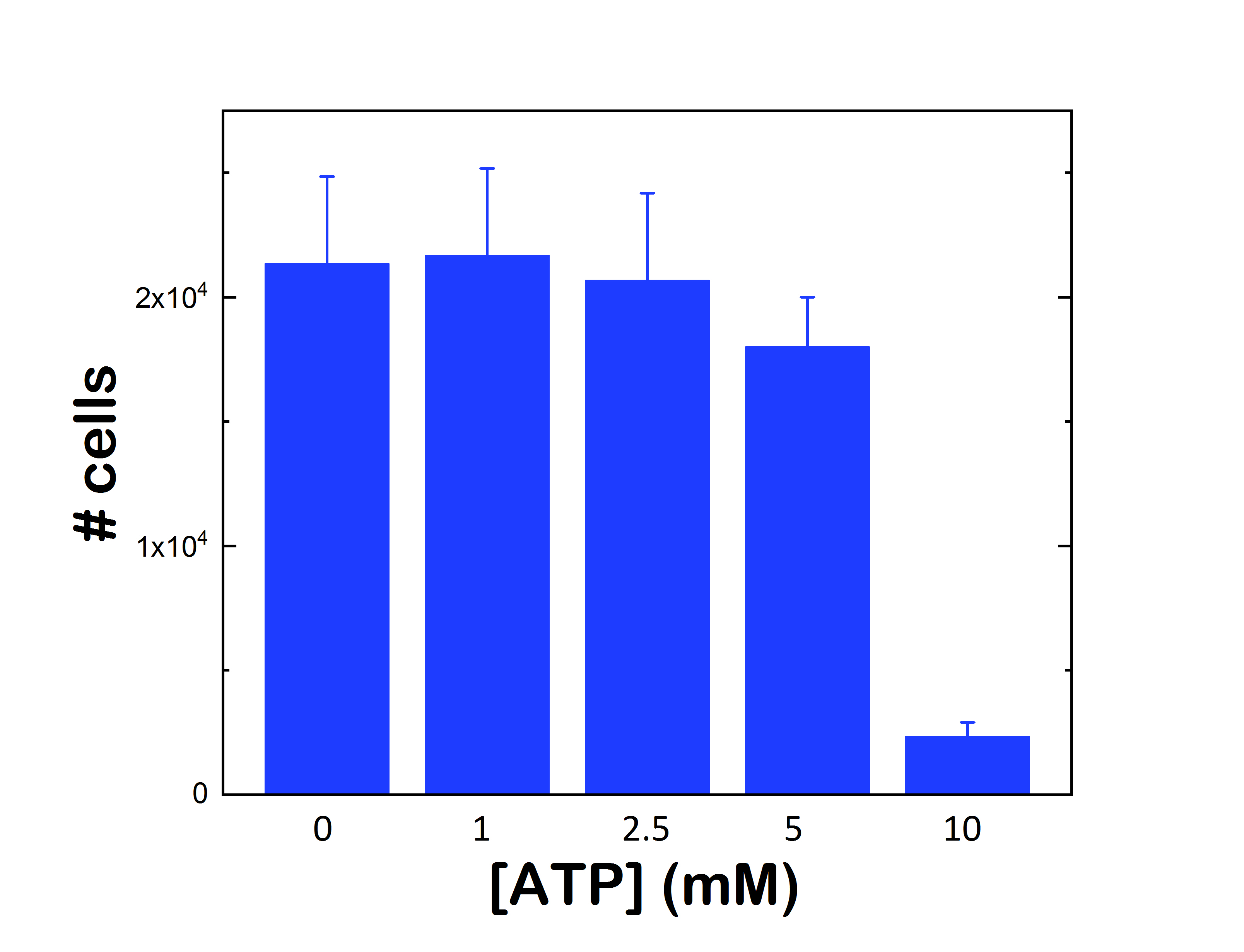


## Figure S3. Cellular viability of mouse embryonic fibroblasts exposed to free ATP. MEFs were incubated with complete DMEM containing to 0 to 10 mM ATP for 24 hours at 37 °C and cell viability is assessed with the Alamar Blue reagent. (See main text for details).


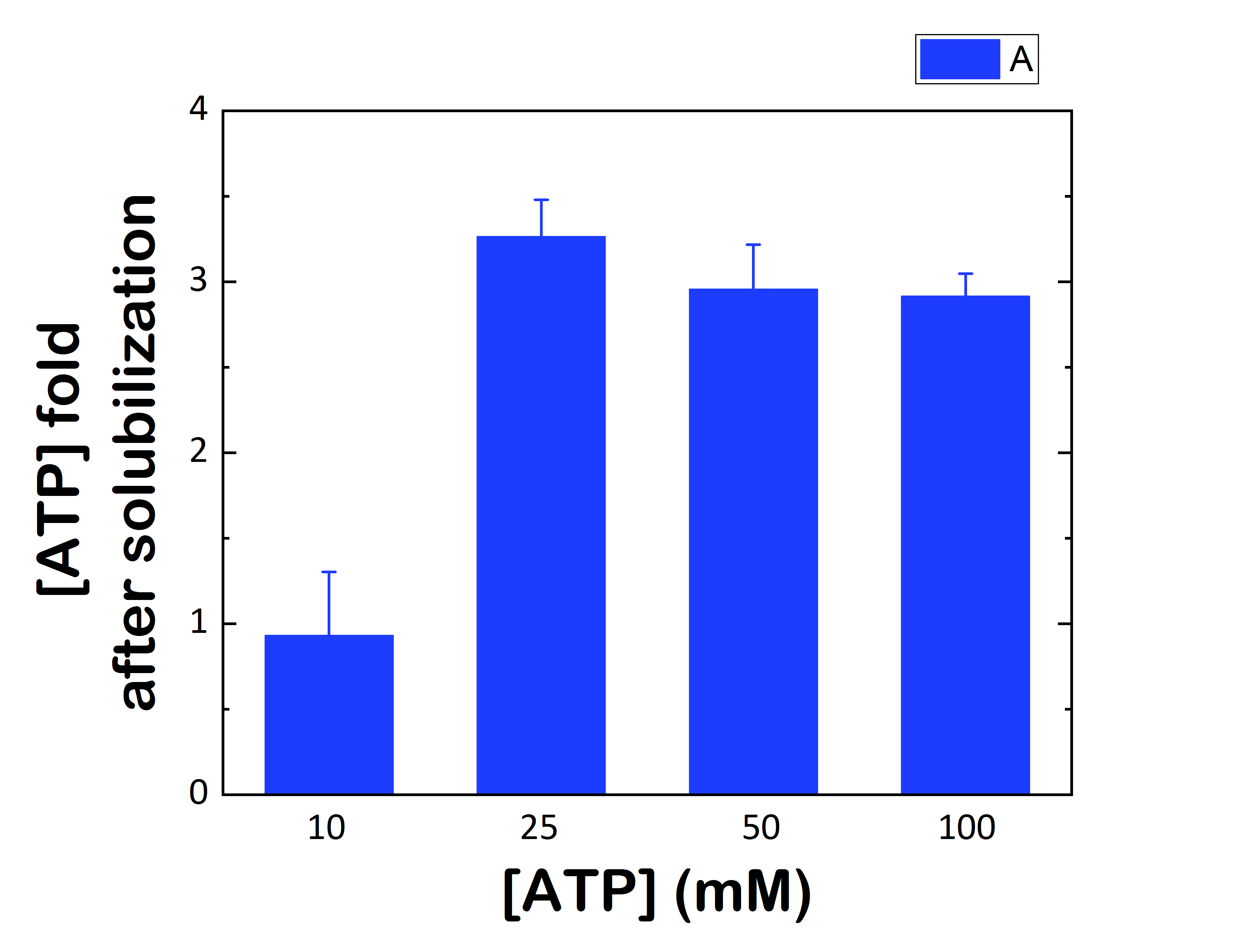


## Figure S4. Minimal concentration of ATP required to be efficiently encapsulated in POPC liposomes carrying 10% mol of DPTE-tbFGF and 10% mol of DPTE-GALA. After encapsulation of increasing amounts of ATP during POPC liposome formation, the liposomes were washed to remove the non-lumenal ATP and then solubilized with 0.1 % Triton-X100 to release the encapsulated ATP. The ATP concentration was then compared to the signal from empty liposomes. The amount of ATP was determined with Luciferin/Luciferase based assay (See Methods for details).
